# Economic support, education and sexual decision making among female adolescents in Zambia: a qualitative study

**DOI:** 10.1186/s12889-021-11372-w

**Published:** 2021-07-09

**Authors:** J. Milimo, J. M. Zulu, J. Svanemyr, E. Munsaka, O. Mweemba, I. F. Sandøy

**Affiliations:** 1 Choma College of Nursing and Midwifery, P.O. Box 630063, Choma, Zambia; 2 School of Public Health, University of Zambia, Ridgeway Campus, P.O. Box 50110, Lusaka, Zambia; 3 CMI - Chr. Michelsen Institute, P.O. Box 6033, N-5092 Bergen, Norway; 4 School of Education, University of Zambia, P.O. Box 32379, Lusaka, Zambia; 5 Department of Global Public Health and Primary Care, Centre for International Health, University of Bergen, Postboks 7804, 5020 Bergen, Norway

**Keywords:** Female adolescent, Early marriage, Economic support, Social cash transfer

## Abstract

**Background:**

The importance of educating female adolescents has been recognized as critical to the development of any country. However, in low income countries like Zambia they often drop out of school due to poverty, early pregnancy and early marriages. Some studies indicate that economic support such as Social Cash Transfers (SCTs) can mitigate the effects of poverty on female adolescents by improving their school participation and helping postpone pregnancy and marriage. This study aimed to explore the role of economic support in influencing education and sexual decision making among female adolescents in a randomised controlled trial in Zambia.

**Methods:**

The study adopted a qualitative approach. It utilized purposive and convenient sampling. Data were collected from 6 schools using 18 in-depth interviews (IDIs) and 4 focus group discussions (FGDs) comprising 48 school-going female adolescents in grade 8 aged 14 to 17. All participants received economic support in form of SCTs and payment of school fees as part of the Research Initiative to Support the Empowerment of Girls (RISE), a Cluster Randomised Controlled Trial. Data were analyzed using thematic analysis.

**Results:**

Findings suggested several benefits of the economic support for the female adolescents such as economic independence and empowerment; increased assertiveness and autonomy; reduced desire for sexual relationships with boys in exchange for cash and gifts; increased motivation for school; enhanced parental and community support for female adolescents’ education and; reduced school dropouts. However, they also experienced jealousy from those who did not benefit from the economic support.

**Conclusion:**

Economic support played a significant role in influencing both educational and sexual decision making among female adolescents.

**Trial registration:**

ISRCTN Registry: ISRCTN12727868, (4 March 2016).

**Supplementary Information:**

The online version contains supplementary material available at 10.1186/s12889-021-11372-w.

## Background

Worldwide 62 million adolescents are estimated to drop out of school every year [1]. A World Bank study from 24 low-income countries revealed that poverty remains the most important factor determining whether a female will continue with her education. On average, only 34% of female adolescents in the poorest quintile households in these countries complete primary school, compared with 72% in the richest quintile households [2]. Studies from low-income countries indicate that female adolescents who quit school early are more likely to marry and become pregnant earlier than those who stay in school [3–7]. Child marriage stifles girls’ educational attainment and is a barrier against employment and economic advancement [8]. In addition, early pregnancy can have consequences on their immediate and long-term health. Pregnancy and child-birth complications were the leading causes of death in 15 to 19 years old female adolescents in 2016 globally [9]. The risks of premature and low birth weight are high in adolescent pregnancies [1, 10, 11], resulting in higher morbidity and mortality for the baby [12–15].

The importance of ensuring that female adolescents are educated to secondary level has been recognized as critical to national development since there is evidence that suggests that educating females yields high economic and social returns [16]. When female adolescents succeed in getting a good secondary education, they develop self-confidence and skills that can have a profound impact on society. In developing countries, the social benefits of women’s schooling are reflected in reduced female fertility rates, improved nutrition for pregnant and lactating mothers and infants, reduced infant mortality rates, and reductions in early marriage and pregnancy, among other benefits [17].

Since their introduction in the 1990s in Latin America (Mexico and Brazil), Social Cash Transfers (SCTs) s have been used as a strategy for reducing poverty and improving human welfare. Today, most countries in Latin America, Asia and Africa have implemented some form of cash transfer program in order to mitigate the effects of poverty for vulnerable groups and encourage investments in human capital (education, health and nutrition) [16–18]. Some of these programs have focused on retaining female adolescents in school [16].

The effects of other kinds of economic support for school have also been studied. In Kenya, female adolescents who were offered free school uniforms were less likely to drop out of school before completing the primary level, and reports by classmates indicated reduced risks of early marriage and childbearing [19]. A Randomised Controlled Trial in Zimbabwe found that offering payment of school fees and free uniforms for female adolescent orphans reduced school dropouts with 80%, and marriage rates with 60% over the next 2 years [20].

In Zambia, 54% of the population live in poverty according to the 2015 Living Conditions Monitoring Survey. In the same survey, 40% of female adolescents were reported to have left school due to lack of financial support; 10% due to early pregnancy and 5% due to marriage [21]. In order to explore how economic support may influence beliefs, attitudes, and norms with regards to education and sexuality, this qualitative study was conducted among female adolescents receiving SCTs and payment of school fees as part of the Research Initiative to Support the Empowerment of Girls (RISE) Trial in Southern Province of Zambia.

## Methods

### Study design, study context and participants

This case study adopted an exploratory qualitative research design and drew participants from female adolescents who were recipients of economic support as part of the Research Initiative to Support the Empowerment of Girls (RISE) trial. RISE was a Cluster Randomised Controlled Trial which measured the effectiveness of economic support consisting of SCTs and payment of school fees on grade 9 completion and early childbearing.

The RISE trial recruited 4900 female adolescents from Southern and Central Provinces. A total of 157 schools from the following 12 districts were selected: Chikankata, Choma, Kalomo, Mazabuka, Monze, Pemba, Chibombo, Chisamba, Kabwe, Kapiri Mposhi, Luano, and Mkushi. The trial had three arms. In the control arm, female adolescents were supported with writing materials only. In the second arm, the female adolescents were given school fees from grade 8 to grade 9, writing materials and a monthly unconditional grant of K30 (approximately US$3 PPP 2016) while their parents also received an unconditional annual grant of K350 (approximately US$35 PPP 2016). In the third arm, the female adolescents were given the same writing materials and economic support, and in addition they were invited to attend youth club meetings every fortnight while their parents were invited to community dialogue meetings every 2 months. The youth club meetings provided comprehensive sexual and reproductive health education to adolescent males and females in and out of school. The community dialogue meetings focused on the benefits of education for adolescent females and the postponement of early pregnancy and marriage. The intervention period ran from September, 2016 to November, 2018 [22].

This study was conducted in RISE sites in three districts of Southern Province: Mazabuka, Monze and Pemba. The districts are generally rural. In addition to government administrative structures, traditional leaders exercise significant power and are recognised by government and subjects as the custodians of culture. The main economic activity in the districts is agriculture while the predominant language used is Chitonga. Rural poverty is quite widespread in the area [7].

### Sampling procedure

Two schools from each of three of the study districts were conveniently selected for the study: Mazabuka, Monze and Pemba, bringing the total number of included schools to six.

Study participants were purposively selected among RISE participants from schools in arm 3. Eighteen participants were selected for IDIs while 30 were invited for 4 FGDs, bringing the total number of participants to 48. A preliminary analysis of the information indicated that saturation had been reached. Table 1 below summarises data on the number of participants who took part in IDIs and FGDs in each district.

**Table 1:** Number of IDI and FGD participants per district

District	School identification	IDIs	FGD participants	Total
Mazabuka	1	–	7	7
	2	–	8	8
Monze	1	5	–	5
	2	5	–	5
Pemba	1	5	8	13
	2	3	7	10
**Total**	**(6 schools)**	**18**	**30**	**48**

### Data collection

A semi-structured interview guide for the purpose of this study was developed by the research team and was used by the first and fourth author in conducting both the IDIs and FGDs (Supplementary file 1). Both of the interviewers were from Southern Province and were able to communicate with participants relatively easily. The minimum duration of IDIs and FGDs was approximately 1 h while the maximum was 2 h.

A voice recorder was used during data collection to enable the researchers to pay particular attention to the participants’ non-verbal communication cues and accurately record what was said. Prior to starting each recording, participants were informed about its use and they all gave verbal consent. During the IDIs and FGDs, field notes were also taken to back-up the voice recordings. The researchers translated and transcribed the recorded audio files from the local language Chitonga to English. The researchers have stored the recorded audio files and transcriptions safely and they will be kept for 5 years.

### Pilot study

In order to test the data collection tools and methods, five pilot IDIs and one FGD were conducted at a primary school in Chibombo district prior to the actual study. The pilot involved female adolescents in their eighth grade taking part in the RISE trial. It was conducted by the first, second and fourth authors.

### Data management/analysis

Collected data were analysed manually using thematic analysis. The data validation process involved familiarization with the data, generating the initial codes, searching for themes among codes, reviewing themes, defining and naming major themes and, finally, producing the field report. Three independent researchers were involved in the process of data analysis: developing and refining the codes and the themes. Themes were cross-checked to ensure they were consistent with collected data and interrelated.

### Ethical considerations

The researchers ensured that the required rigorous procedural, ethical and methodological standards for research with minors were followed. To assure anonymity and confidentiality, names were not recorded. For a detailed narrative of specific ethical considerations applied in the study, refer to the Annexes section of the Manuscript under Declarations: Ethical approval and consent to participate - Ethical Considerations.

## Results

### Demographic characteristics of participants

All the 18 IDI and 30 FGD participants were female adolescents aged between 14 and 17 years. All the participants were in grade 8, not married, not pregnant and none had children. Of the 18 IDI participants, 10 lived with both parents; 3 lived with their grandparents; 2 lived with their uncle or aunt; another 3 lived with their single mothers. Eleven had parents/guardians whose main occupation was farming while 7 had parents/guardians who were dependent on casual jobs.

Of the 30 FGD participants, 18 lived with both parents; 6 lived with their grandparents; 3 lived with their uncle or aunt and 3 lived with their single mothers, 15 had parents whose main occupation was farming while another 15 had parents who were dependent on casual jobs.

### Emerging themes

From the IDIs and FGDs, codes, sub-themes and themes emerged as shown in Table 2.

**Table 2:** Codes, sub-themes and themes

Codes	Sub-themes	Themes
• Concentration on education. • Hope for a bright future. • Increased parental support for education.	• Benefits of adolescent females’ education.	• Increased motivation to get education.
• Increased assertiveness and autonomy. • Less dependence on boys. • Reduced desire for sexual relationships with boys for cash and gifts.	• Sexual decision making. • Economic empowerment.	• Reduced dependence on boyfriends and parents.
• Supportive atmosphere. • Unsupportive atmosphere.	• Experience with community, relatives and friends.	• Social dynamics in RISE intervention.

### Increased motivation to get education

The interviews and FGDs suggested that the motivation to get educated among female adolescents in the communities was generally low. Most participants reported that with the economic support rendered as part of the RISE trial, they now felt encouraged to continue with school. Participants mentioned that education beyond primary level was important for them. One participant had this to say:

*“For now I want to concentrate on my education so that I go to college and start working in future. I want to help my family when I start working”,* IDI, P2 (S3).

Participants reported that if they did not get educated, they were likely to get married early, hence the need for them to concentrate on education and later start working. They claimed that female adolescents if educated would help the parents, siblings and the community in the future when they start working. They believed that boys would only help the relatives to their wives. One of the participants said:

*“It is good for a girl-child to learn so that she does not suffer in the future, especially with so many problems nowadays where parents cannot even manage well to provide for their families; it is easy when the girl child is working as she can take care of her parents as well as her own children”,* FGD, P5 (S1).

The participants stated that many female adolescents without good education that get married are prone to suffering as they are dependent on their partner’s support for all. This makes women vulnerable and at risk of abuse by their partners. One of the participants narrated:

*“It is good for girls to learn so that they have a bright future; some women do suffer a lot in marriage. For example, my auntie is always abused physically by my uncle for no reasons or over useless things. It is good for girls to be educated and later start working”,* IDI, P3 (S4).

Previously, parents were more in support of adolescent males’ education as females were perceived to be at risk of getting married at any time and dropping out of school. However, the majority of the participants said that their parents’ attitudes towards female adolescents’ education had changed after the RISE Project started, because they had realised that girls would be capable of taking good care of them in future. In relation to this, one participant said the following:

*“Previously our parents were only concerned with our brothers’ education, but now my parents are concerned with my education. My mum is always checking what I have learnt at school every day, and may even beat me if I haven’t done my homework”,* FGD, P2 (S1).

The study established that not only the parents were in support of girls’ education, but also other family members, including grandparents. In addition, family members discouraged adolescent females from sexual relationships and helped them, where possible, to focus on school work:

“*My family is happy with girls’ education; at night my grandmother gives me a torch to help me with my studies. She encourages me to abstain from sexual relationships with boys and instead to concentrate on my education”,* IDI, P1 (S2).

### Reduced dependence on boyfriends and parents

Most of the participants narrated that female adolescents are easily influenced by fellow peers to engage in sexual relationships at an early age in order to access money and gifts from boys. Some participants said that they used to request for pocket money from their parents. They felt excited about the pocket money given to them from the RISE Project because they were free to use their money in any way they wanted. According to them, it had reduced the desire to get a boyfriend and made them reject sexual relationships with boys. The pocket money was even more than what the boys used to give them. One of the participants narrated:

*“I have money; I don’t need a man, for what? I told off one man who was proposing love to me; I told him I don’t need a man; I have money already. This money has given us power to reject sexual proposals from men”,* IDI, P2 (S4).

Another participant narrated:

“*I now have the power to reject sexual proposals on my own without even refusing through someone else. I decide on my own because I want to learn, I already have money. I don’t want to die while giving birth or due to pregnancy related complications since am still too young to fall pregnant”,* IDI, P3 (S6).

One of the participants explained that she had even been empowered to end a relationship:

*“My boyfriend used to give me money. When RISE came in, I had to quit the relationship because I had money from RISE, although my boyfriend still wanted us to continue with the relationship. I never loved him, I just wanted his money. He used to give me K20.00* (approximately US$2 PPP 2016) *so I compared with what I was receiving from RISE. Then I thought of quitting the relationship so that I now concentrate on school”,* IDI, P3 (S4).

One of the participants revealed that the money helped them focus on school and narrated:

*“When we get this money, we do not even think about boys and we do not even trouble our parents for money. We remain focused on our school ....it is easy to think about getting married when you are not in school”,* FGD, P6 (S5).

However, despite receiving the SCTs, not all participants changed their behaviour of having boyfriends. One of the participants had this to say:

*“Some girls even after being under RISE still have boyfriends; they are just influenced by their friends to fall in love with some boys in order to be given more money”,* FGD, P4 (S1).

### Social dynamics in RISE intervention

Most of the participants said that their siblings and relatives were happy with the support given to them by RISE. The participants reported that most of their relatives congratulated them for the support and encouraged them to work hard in school as they were assured of support from RISE. One of the participants stated:

*“Others do appreciate the help that we receive. My friends tell me that at least RISE is helping me and has even taught me good morals. It will help me not to drop out of school”,* FGD, P2 (S1).

Most participants reported that the majority of community members were happy with the economic support given to the female adolescents and were now encouraging them to concentrate on their education for a bright future instead of engaging in sexual relationships with boys. However, according to the other participants, some of their friends and siblings felt that the female adolescents should share the money or snacks with them all the time.

After witnessing the benefits of SCTs and the payment of school fees, the participants reported that their parents now believed that investing in female adolescents’ education would prevent poverty. According to participants, most parents hoped that education with some kind of skills training would enable female adolescents to run some business to sustain their lives and that of their families.

However, according to some of participants, the economic support contributed to an unsupportive social atmosphere for them. For instance, some of their friends, especially adolescent males, ridiculed them when they received their money. The participants revealed that boys were offering to give them more money than what they were receiving from RISE. They also reported that adolescent males were angry because their sexual proposals were rejected due to the pocket money from RISE. They accused participants of being too proud and accepting evil money. One participant reported:

*“Some boys even say that we are receiving money from people we don’t know and we are not even sweating for it. They say that we shall see what will happen when that money stops flowing. That is when their money will start flowing again”,* FGD, P5 (S3).

Some community members also spoke negatively about the economic support the female adolescents were receiving from RISE. They accused them of being prostitutes and wished them pregnant so that they could stop school. Some community members associated every monetary gift to Satanism or prostitution as one participant narrated:

*“Some villagers say it is satanic money, but we usually tell them that it is not satanic money; it is money from University of Zambia to help girls learn. Others say the money is just influencing the girls into prostitution because they are given money by boys and they cheat that it is from RISE”,* IDI, P3 (S6).

Due to high prevalence of poverty in the community, there were some expressions of negative emotions and jealousy-talk from some of the female adolescents’ relatives, friends and community members each time they received their pocket money. This made some participants feel uncomfortable even if they used to share the few snacks they bought using RISE money. One of the participants narrated this:

*“One of my uncles passed his exams going to grade 10 but our parents have no money to send him to school. So as he sees me going to school every day, he feels jealousy, he says that my parents are just favouring me to be in school alone, I feel bad because it normally brings a lot of talking at home”,* FGD, P1 (S2).

## Discussion

The study indicated that female adolescents receiving economic support from the RISE project felt that the support helped them concentrate on education and that it had strengthened their belief that employment will be a major benefit of their education. Before the start of the RISE trial, education for male adolescents was given more priority in the community than that of females. Participants reported that with the economic support, their parents tended to encourage them to learn and focus on education. Parents and relatives now believed that education would enable female adolescents to help them and the community in the future when they get employed. However, the fact that only a few female adolescents received the economic support created envy in the community and sometimes within the family.

Participants indicated that economic support made them more assertive when rejecting sexual advances from males, more resistant to bad advice and able to focus on education. Economic support had empowered them to quit sexual relationships. Other studies support these findings that economic support can lead to economic empowerment of women in general and, female adolescents, in particular [16, 23]. Temin and colleagues [24] argue that SCTs give female adolescents the means to decide on their own lives, and to plan for their future, thereby triggering hopefulness. These skills can also produce other positive outcomes such as HIV prevention and reduction in social and economic inequality [25].

According to participants, SCTs and the payment of school fees led to improved school attendance and helped a lot of adolescent females remain in school, partly due to removal of economic barriers, but possibly also due to enhanced behavioural control to refrain from engaging in sex and to concentrate on learning. It is likely that the guardians perceived the economic support to be a golden opportunity for their daughters to lead a better life, hence they wanted them to exploit it as much as possible. This is in line with other studies which have found that economic support in the form of social cash transfers successfully improved school attendance in Brazil, Colombia, Mexico, Nicaragua and Zambia [17, 18, 26–28] . However, it is possible that the community dialogue meetings attended by participants also could have led to increased parental support for female adolescents’ education which, in turn, might have influenced school attendance positively and reinforced the effects of the economic support.

Study results suggest that some community members discouraged participants from continuing to secondary school and encouraged them to get married and bear children at an early age. Such bad advice was possibly due to jealousy of the economic support the female adolescents received. This illustrated how the economic support appeared to generate both supportive and divisive environments for girls within their communities. This is supported by reviewed literature by Bastagli and colleagues [26] from their rigorous review of 165 studies on the impact of 56 social cash transfer programs from 30 low- and middle-income countries worldwide from 2000 to 2015. They argue that while economic support has several positive outcomes, there is a risk that social cash transfers can invoke negative feelings in a community. The extent to which such effects are triggered by economic support, may be strongly linked to the design and implementation features of programs. To avoid such community feelings and make them more acceptable, economic support interventions should have a deliberate and effective communication strategy and formulation of local committees in order to ensure that the selection of beneficiaries is regarded as legitimate. Negativity can also be mitigated through making economic support universal in identified poor communities so that all vulnerable children can access it. This was not done in the RISE trial because it was a research project aiming to test an intervention for a limited period in one cohort of female adolescents.

### Strengths and limitations of the study

There are several strengths and limitations associated with the qualitative approach adopted for the study. Researchers gathered rich information by actually talking to participants and relevant others directly, observing their behaviour directly in a face-to-face interaction. The researchers’ personal background, culture and experiences coming from Southern province themselves enhanced their ability to interpret the findings and attach meaning to what was said [29]. The trustworthiness of interpretations was ensured by constantly cross-checking codes and refining themes by independent reviewers.

Other limitations were that only views expressed by female adolescents were studied. Views of adolescent boys’ and of other community members were not explored. Another limitation is that the information gathered by the study was self-reported and only obtained from one of the intervention arms in the RISE trial (the third arm). Researchers did not verify the claims of the participants by checking school attendance registers or by comparing the participants’ behaviours with those of participants in the control arm who did not receive the same type of intervention. The participants interviewed in this study were also receiving other forms of support than SCTs from RISE. Therefore, some of the effects reported by the participants might have been due to other components of the intervention package, such as the impacts of youth clubs, or community meetings, or the payment of school fees. Further, the role of the two researchers who conducted the interviews may also have influenced the results. The participants may have felt compelled to report positive effects because they may have perceived the interviewers to be part of the RISE team. Although they shared the same tribe and language with the participants, the interviewers were much older than the female adolescents, more educated, one was male, and neither of them came from the study areas.

While the findings of this study may not automatically be generalized to other settings, the findings may be of interest to other researchers and institutions interested in the effects of SCTs and other economic support for female adolescents in similar poor settings in the sub-region.

## Conclusion

Economic support, such as SCTs, played a significant role in influencing both educational and sexual decision making among female adolescents in this study. Specifically, economic support enhanced hope for a bright future for female adolescents and their families. It also enhanced the female adolescents’ assertiveness and significantly reduced their dependency on boys for gifts and money in exchange for sex. Ultimately, economic support can help prevent STIs, early pregnancies and early marriages among female adolescents while maintaining them in school. Notwithstanding, economic support also generated negative ramifications such as envy among community members who did not benefit from it.

## Supplementary Information

**Additional file 1.** Interview and discussion guide for girls on social cash transfer under the RISE project. * Description of data: Interview and discussion guide.

## Data Availability

The data can be obtained from the authors on reasonable request.

## Abbreviations

CCT: Conditional Cash Transfer
DFID: Department for International Development
RISE: Research Initiative to Support Girls’ Education
UCT: Unconditional Cash Transfer
UN: United Nations
UNFPA: United Nations Population Fund
UNICEF: United Nations Children Emergency Funds
WHO: World Health Organization
