# Supplementary material for: Economic support, education and sexual decision making among female adolescents in Zambia: a qualitative study
Source: BMC Public Health. 2021 Jul 9;21:1360. doi: 10.1186/s12889-021-11372-w (PMC8268295; doi:10.1186/s12889-021-11372-w)
Supplement: Supplementary file 1 — Additional file 1. Interview and discussion guide for girls on social cash transfer under the RISE project. * Description of data: Interview and discussion guide. [file 12889_2021_11372_MOESM1_ESM.docx]

### INTERVIEW AND DISCUSSION GUIDE FOR GIRLS ON SOCIAL CASH TRANSFER UNDER THE RISE PROJECT

**Introduction:**

Good morning/afternoon, my name is Joyce Milimo. I am a student in the school of public health at University of Zambia in Lusaka. I am conducting a study on the Role of Social Cash Transfer in influencing Educational and Sexual decision-making among Girls in Monze and Choma districts. The information I will collect will help us come up with programmes aimed at improving girls’ education in this country. I am therefore, inviting you to participate in this study.

**Demographic characteristics**

Briefly tell me about yourself

**Find out about**

1. Age
2. Current grade in school
3. Marital status
4. Number of children if any
5. If pregnant
6. Guardian
7. Who are you living with: Parents, sisters or brothers, others?
8. Could you tell me what your parents do for a living?

**Experience with social cash transfer in relation to education, relationships and sexuality**

**Find out about;**

1. In your own view, would you explain to me why you think it is important for girls to go to school?
2. Tell me what you think about the economic support that you have been receiving?
3. You have received some pocket money. How important has this money been to you?
4. How have you spent the money? (Probe: Snacks, food, clothes, etc.)?
5. Before you started receiving the cash transfer, how were you accessing pocket money? (Probe: From parents, boy friend, relatives etc)?
6. What challenges, if any, have you faced when using the money you have been receiving for upkeep? (Probe: are you required to buy food for the family, is it enough to meet your needs?)
7. How has the cash transfer affected your relationship with your parents, siblings, members of the opposite sex and friends?
8. Would you tell me how the cash transfer that you receive has affected decision making in your family as well as conflicts over money?
9. What would you say has been the impact of the money that has been given to girls on reducing unwanted pregnancies, early marriages, and school dropout among school girls in this community?
10. How has the cash transfer support you are receiving affected your family’s attitude towards girls’ education?)
11. We know that many adolescent girls are going out with boys and men to get help to buy snacks and other things. In your view, how has the cash transfer affected this tendency by girls to go out with boys and men for money and other things? (Probe: Has the cash transfer helped to stop girls from engaging in sexual relationships with boys for cash and gifts?).
12. In which ways has the money you have been receiving given you power to decide for yourself in sexual matters?
13. In your view, what has been the community’s attitude towards the financial help given to (Probe: girls, families to support their children’s education?)
14. Tell me about your dreams and ambitions (Probe: who/what has been your source of inspiration?)
